# Clinical Studies on the Treatment of Novel Coronavirus Pneumonia With Traditional Chinese Medicine—A Literature Analysis

**DOI:** 10.3389/fphar.2020.560448

**Published:** 2020-09-10

**Authors:** Zhihuan Zhou, Ning Gao, Yumeng Wang, Pengcheng Chang, Yi Tong, Shufei Fu

**Affiliations:** ^1^ College of Traditional Chinese Medicine, Tianjin University of Traditional Chinese Medicine, Tianjin, China; ^2^ Graduate School, Tianjin University of Traditional Chinese Medicine, Tianjin, China; ^3^ Clinical Department, First Teaching Hospital of Tianjin University of Traditional Chinese Medicine, Tianjin, China

**Keywords:** novel coronavirus pneumonia, traditional Chinese medicine, clinical research, Drug application rule, literature analysis

## Abstract

**Objective:**

This study aims to analyze the current situation and characteristics of traditional Chinese medicine for treatment of novel coronavirus pneumonia, clarify its clinical advantages and provide a reference for clinical treatment.

**Methods:**

Clinical randomized controlled trials, clinical control trials and case series research involving the use of Chinese medicine for novel coronavirus pneumonia treatment were selected from PubMed, Chinese Journal Service Platform of CNKI, VIP, and WanFang Data Knowledge Service Platform from the establishment of the library to 11:00 am on April 15, 2020. The published information, research design, intervention measures and research observation index were statistically analyzed.

**Results:**

Twenty studies were included. The research methods were mainly clinical controlled trials. The observation indicators were mostly fever improvement time, cough improvement time, shortness of breath improvement time, chest CT and CRP examination. Maxing Ganshi (*Ephedrae Herba, Armeniacae Semen Amarum, Glycyrrhizae Radix Et Rhizoma*, and *Gypsum Fibrosum*) decoction was the core prescription. The most frequently used drugs were *Glycyrrhizae Radix Et Rhizoma* (Gancao)*, Ephedrae Herba* (Mahuang)*, Armeniacae Semen Amarum* (Kuxingren)*, Atractylodis Rhizoma* (Cangzhu), and *Scutellariae Radix* (Huangqin). The most frequently used drug combination was *Ephedrae Herba* (Mahuang)–*Armeniacae Semen Amarum* (Kuxingren). The most frequently used Chinese patent medicine was Lianhua Qingwen capsule/granule.

**Conclusions:**

Traditional Chinese medicine has widely used for novel coronavirus pneumonia in China. It is worthy of global attention. Also, high-quality randomized controlled clinical trials on the effectiveness and safety of traditional Chinese medicine in the treatment of novel coronavirus pneumonia need to carry out.

## Introduction

Recently, new coronary pneumonia (NCP) outbreaks worldwide, according to the daily information released by the Chinese State and Regional Health Committees’ daily information as of 21:31 on April 16, 2020, China has confirmed a total of 83,798 cases and 3,352 cumulative deaths; among the cumulative confirmed cases of 2,019,857 worldwide, 135,165 died and 1,422,853 remained infected (Dingxiangyuan, 2020). The epidemic trend in regions outside of China has greatly erupted, overseas outbreaks have escalated, and more than 20 countries and regions have been infected. Except for Antarctica, all continents have confirmed cases. How to effectively treat NCP remains a key problem. The Office of the State Administration of Traditional Chinese Medicine and the General Office of the National Health And Health Commission have issued seven editions of the “Diagnosis and Treatment Plan of Novel Coronavirus Infection Pneumonia”; each version of the diagnosis and treatment plan has always emphasized the active role of Chinese medicine in the treatment and the strengthening of its combination with Western medicine to promote medical treatment and achieve good results (National Health Commission of the People’s Republic of China, 2020). In an interview, Zhong Nanshan affirmed the role of Chinese medicine in treatment of NCP; Chinese medicine can effectively suppress inflammatory damages and can also be popularized in foreign countries (Tencent News, 2020a). The article aimed to systematically organize clinical research by literature metrology and data mining methods, analyze the current situation of clinical treatment research in Chinese medicine, explore the clinical treatment characteristics of Chinese medicine and provide a reference for global clinical treatment of NCP.

## Materials and Methods

### Search Strategy

Two reviewers (ZZ and NG) independently isolated the useful information from the database. Studies that used Chinese medicine to treat NCP were selected from PubMed, Chinese Journal Service Platform of CNKI, VIP, and WanFang Data Knowledge Service Platform. Advanced search was conducted using the following terms: “NCP” or “Novel Coronavirus Infection” or “New Coronavirus” “2019-nCoV” “COVID-19” “SARS-CoV-2” containing “Chinese and Western medicine” or “Chinese medicine” or “Traditional Chinese medicine” or “prescription.” The search time was from the establishment of the library to 11:00 on 15 April 2020.

### Inclusion and Exclusion Criteria

Inclusion criteria: All studies on clinical treatment of NCP in Chinese medicine that state complete treatment options and processes and are classified as clinical control trials (CCT), randomized controlled trials (RCT), and case series studies (CS) were included.

Exclusion criteria: Studies categorized as review, basic research, regional epidemiological research, experience summary, and syndrome analysis were excluded.

### Data Extraction and Analysis

Noteexpress, a document management software program, was used to manage the studies obtained from different databases. An access database was established to extract information on the publication of the literature (author, time of issue, issue journal, type of fund), research design (number of cases, subject gender and age), intervention measures (prescription, traditional Chinese medicine), research observation indicators and other information for statistical analysis. For eligible studies, two review authors (ZZ, and GN) extracted the data independently. Disagreements were resolved through consultation with a third party (FS).The law of the prescription use of Chinese medicine was analyzed statistically through the “Traditional Chinese medicine inheritance auxiliary system.”

## Results

### Description of Studies

**Figure 1:**
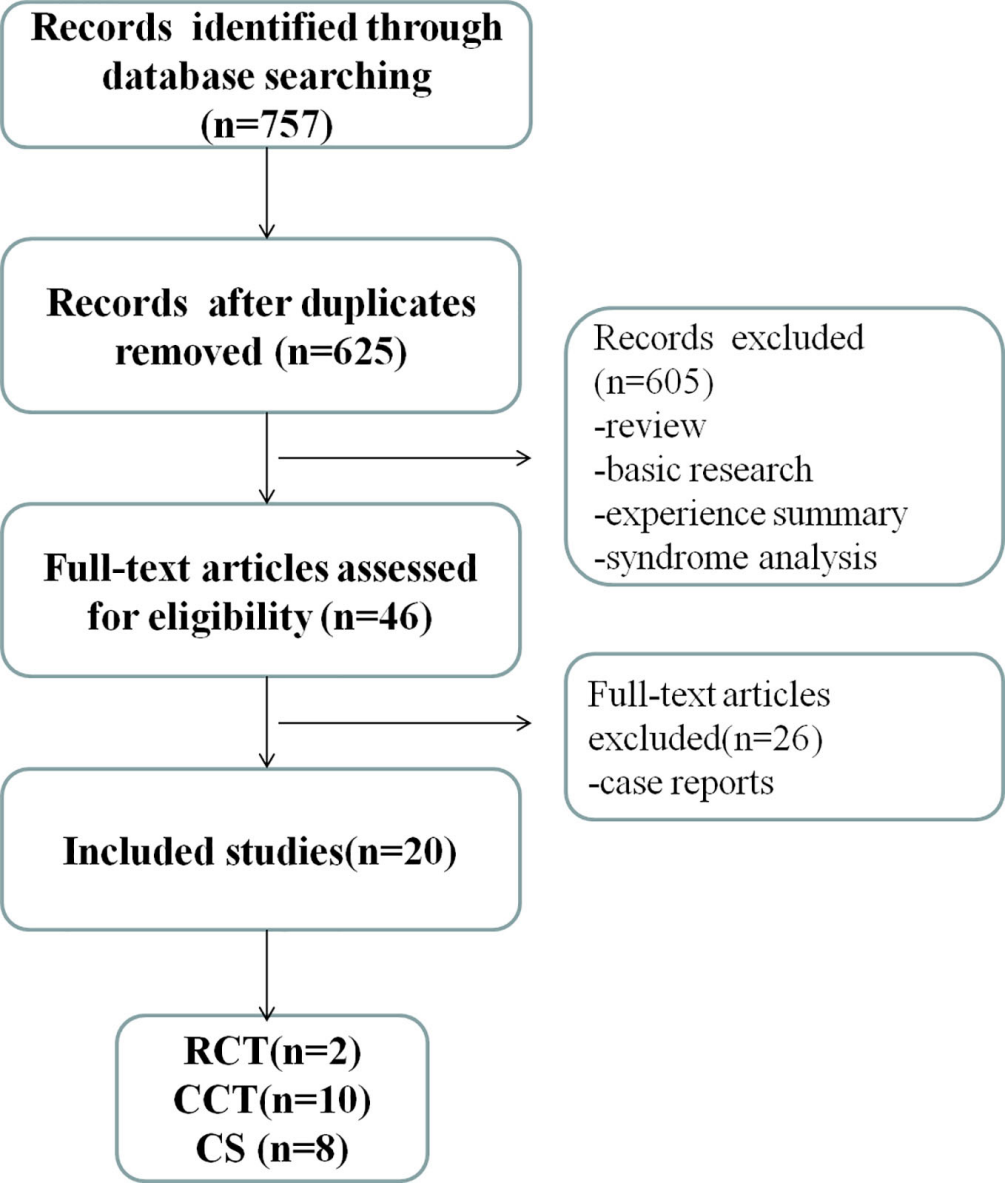
Literature screening process and results.

We identified 757 potentially relevant articles. After removal of duplicates, 625 records remained. After going through the titles and abstracts, we exclude 605 papers. By reading the full text of the remaining 46 articles, 26 were exclude because they were case reports. Ultimately, 20 studies were included in present study (Bin et al., 2020; Cheng and Li, 2020; Cheng et al., 2020; Ding et al., 2020; Duan et al., 2020; Fang et al., 2020; Fu et al., 2020; Gong et al., 2020; Hu et al., 2020; Lv et al., 2020; Qv et al., 2020; Shi et al., 2020; Wang Y. et al., 2020; Wang T. et al., 2020; Xia et al., 2020; Xiao et al., 2020; Yang Q. et al., 2020; Yang Z. et al., 2020; Yao et al., 2020; Zhu et al., 2020). Among these studies, 2 RCTs, 10 CCTs and 8 CSs were included, which accounted for 10.00%, 50.00% and 40.00% of the total number of studies, respectively. The specific screening process is shown in **Figure 1**.

### Basic Characteristics of the Literature

The basic characteristics of the 20 trials are summarized in **Tables 1** and **2**. The first study on clinical treatment involving Chinese medicine for treatment of NCP was published on February 6, 2020 (Gong et al., 2020). After February 15, the volume of studies published began to increase. By March 25, 19 articles were published. By April 4, the volume of literature published showed a downward trend. The total number of observations was 1,810, of which 1,021 and 789 were males and females, respectively. The age ranged from 0.6 to 95 y. The largest number of subjects in the study was 308 (Wang T. et al., 2020), and the minimum number of study cases was 13 (Cheng and Li, 2020). About the research areas, the worst-affected area, Hubei region, had the largest volume of studies, accounting for more than 50%, followed by Henan and Anhui regions. 12 trails were funded by research projects. All trials adopted decoction or patent medicine of traditional Chinese medicine (TCM) therapy combination with western treatment in the trial group for NCP. While the control group only adopted western treatment. For the severity of included subjects, most RCTs and CCTs included subjects who were the mild or common type, while the subjects in CS were common type and serious type. Three studies mentioned death cases (Bin et al., 2020; Xia et al., 2020; Yang Q. et al., 2020). One study mentioned there were no death cases (Wang T. et al., 2020). The other 16 studies did not mention the death condition. Adverse reactions were reported in eight studies, while no mention in the other studies. Specific research characteristics of RCTs and CCTs are shown in **Table 1** and CSs are shown in **Table 2**.

**Table 1:** Basic characteristics of the included studies (RCT and CCT).

Included trials	Funding	Study designs	Study region	Sample characteristics type; male/female; age(y)		Interventions		Duration	Fever improvement time(d)	Outcome index	Intergroup differences	Adverse reactions
				Trial	Control	Trial	Control					
YAO 0206 (Yao et al., 2020)		CCT	Hubei	CT:21 M: 16, F: 5 57.1 ± 14.0	CT: 21 M: 12, F: 9 62.4 ± 12.3	Chinese patent drug+WT1.2.6.7	WT1.2.6.7		T: 4.6 ± 3.2 C: 6.1 ± 3.1	1. Disappearance rate of fever and cough 2. Disappearance rate of fatigue 3. Fever improvement time 4. Disappearance rate of anhelation, expectoration 5. Disappearance rate of sore throat, choking sensation in chest, dyspnea, headache, nausea, anorexia, diarrhea, muscle pain 6. Death rate	1.P<0.05 2.P>0.05 3.P>0.05 4.P<0.05 5.P>0.05 6.Not mentioned	
	
LV 0217 (Lv et al., 2020)		CCT	Hubei	MT, CT: 63 M: 28, F: 35 59.1 ± 15.61	MT, CT: 38 M: 18, F: 20 60.2 ± 17.01	Chinese patent drug +WT1.2.3.5.7.8	WT1.2.3.5.7.8	10 d	T: 6 (median) C: 7 (median)	1. Disappearance rate of fever, fatigue, cough 2. Disappearance rate of anhelation, moist rale 3. Fever improvement time 4. Disappearance rate of muscle pain, expectoration, nasal obstruction, nasal discharge, sore throat, choking sensation in chest, dyspnea, headache, nausea, vomiting, anorexia, diarrhea 5. Aggravation rate 6. Death rate	1.P<0.05 2.P<0.05 3.P>0.05 4.P>0.05 5.P>0.05 6. Not mentioned	No adverse response
	
XIA 0218 (Xia et al., 2020)	√	CCT	Hubei	CT: 27 ST: 7 M: 17, F: 17 54.18 ± 13.08	CT: 13 ST: 5 M: 6, F: 12 53.67 ± 12.70	decoction+WT1.2.7.8	WT1.2.7.8	7–10 d	T: 2.64 ± 1.31 C: 4.38 ± 1.90	1. Fever improvement time 2. Recovery time of cough, fatigue, dyspnea, diarrhea) 3. Score of TCM syndrome scale 4. Incidence of mild type to severe type 5. Improvement rate of lung CT 6. Death rate	1.P<0.01 2.P<0.01 3.P<0.05 4.<0.05 5.P>0.05 6. Trial 0%; Control 5.6%	No adverse response
	
QU 0226 (Qv et al., 2020)	√	CCT	Anhui	MT, CT: 40 M: 25, F: 15 40.65 ± 8.23	MT,CT:30 M: 16, F: 14 39.82 ± 6.40	Chinese patent drug+WT1.2	+WT1.2	10 d	T:3.24 ± 0.89 C:5.10 ± 1.40	1. Improvement time of temperature, dry cough, nasal obstruction, Fever improvement time, sore throat, fatigue, diarrhea 2. Dime of nucleic acid test turning negative 3. Death rate	1.P<0.05 2.P<0.05 3. Not mentioned	Trail: 1 case of nausea; Control: 2 cases of nausea
	
DING 0303 (Ding et al., 2020)		RCT	Hubei	MT: 10 CT: 36 ST: 5 M: 39, F: 12 54.7 ± 21.3	MT: 11 CT: 34 ST: 4 M: 39, F: 10 50.8 ± 23.5	decoction+WT1.2.6	WT1.2.6	10 d		1. Disappearance rate of fever, cough, choking sensation in chest and anhelation 2. Disappearance rate of nasal obstruction, abdominal pain, and diarrhea 3. Improvement rate of ESR 4. Improvement rate of CRP, IL-6 5. Improvement rate of TNF-γ, TNF-α 6. Improvement rate of lung CT 7. Liver function 8. Death rate	1.P<0.05 2.P>0.05 3.P<0.01 4.P<0.05 5.P>0.05 6.P<0.05 7.P>0.05 8. Not mentioned	
	
SHI 0305 (Shi et al., 2020)	√	CCT	Shanghai	MT: 1 CT: 40 ST: 8 M: 26, F: 23 47.94 ± 14.46	MT: 1 CT: 14 ST: 3 M: 10, F: 8 46.72 ± 17.40	Chinese patent drug+decoction+WT1.2.3.8	WT1.2.3.8	6 d	T: 16 (4,42) C: 17.5 (8,42)	1. Clinical syndrome integral 2. Hospitalization time 3. Course of disease, fever improvement time 4. Improvement rate of lung CT 5. Death rate	1.P<0.05 2.P<0.05 3.P>0.05 4.P>0.05 5. Not mentioned	
	
XIAO 0310 (Xiao et al., 2020)		CCT	Hubei	MT: 100 M: 64, F: 36 60.90 ± 8.70	MT: 100 M: 66, F: 34 62.20 ± 7.50	Chinese patent drug+WT1	WT1	2 w	T: 2.25 ± 1.12 C: 3.08 ± 1.64	1. Total effective rate 2. Lung CT 3. Fever improvement time 4. Disappearance time of cough, fatigue, dizziness, nasal discharge 5. WBC, Lymph% 6. Death rate	1.P<0.05 2.P<0.05 3.P<0.05 4.P>0.05 5.P<0.05 6. Not mentioned	Trail: 1 case of drug allergy: 2 cases of abdominal pain and diarrhea; Control: 2 cases of drug allergy, 1 case of abdominal pain and diarrhea
	
CHENG 0311 (Cheng et al., 2020)		CCT	Hubei	CT: 51 M: 26, F: 25 55.5 ± 12.3	CT: 51 M: 27, F: 24 55.8 ± 11.6	Chinese patent drug+WT1.2.8	WT1.2.8	7 d	T:2.9 ± 1.7 C:3.9 ± 1.3	1. Disappearance rate and time of fever, fatigue, cough 2. Effective rate of main symptoms 3. Disappearance rate of expectoration, anhelation, choking sensation in chest, anorexia 4. Disappearance rate of muscle pain, dyspnea, nausea 5. Improvement rate of lung CT 6. Rate of turn to severe type 7. Death rate	1.P<0.05 2.P<0.05 3.P<0.05 4.P>0.05 5.P>0.05 6.P<0.05 7. Not mentioned	
FU 0320 (Fu et al., 2020)	√	CCT	Hubei	CT: 37 M: 19, F: 18 45.26 ± 7.25	CT: 36 M: 19, F: 17 44.68 ± 7.45	Chinese patent drug+WT1.7	WT1.7	10–15 d		1. Accumulated points of fever, cough, dry throat and sore throat, choking sensation in chest and anhelation, fatigue 2. Effective rate, hospital discharge rate 3. Absolute value of LYM, CRP 4. WBC, LYM ratio 5. Death rate	1.P<0.05 2.P<0.05 3.P<0.05 4.P>0.05 5. Not mentioned	No adverse response
	
WANG 0323 (Wang Y. et al., 2020)	√	RCT	Hubei	MT, CT: 10 M: 5, F: 5 54.90 ± 3.71	MT,CT:10 M:5.F:5 55.90 ± 3.71	decoction,incense+WT1.	WT 1.2.8.	7 d		1. Clinical symptoms improved conditions (fatigue, cough, dry throat, short of breath) 2. Lung CT 3. Nucleic acid test turning negative 4. Death rate	1.P<0.05 2.P>0.05 3.P>0.05 4. Not mentioned	
	
DUAN 0324 (Duan et al., 2020)	√	CCT	Hubei	MT: 82 M: 39, F: 43 51.99 ± 13.88	MT:41 M:23.F:18 50.29 ± 13.17	Chinese patent drug+WT1.2.6.7	WT1.2.6.7	5 d		1. Disappearance condition of fever 2. Disappearance time of fatigue, cough, expectoration, diarrhea 3. Disappearance time of aversion to cold, bodily pain, sore throat, pharyngalgia, dry throat 4. Score of TCM syndrome scale 5. Hamilton Anxiety Scale 6. Death rate	1.P<0.01 2.P<0.05 3.P>0.05 4.P<0.01 5.P<0.01 6. Not mentioned	Trail: 27 cases of diarrhea Control: no adverse response
	
YANG 0414 (Yang Z. et al., 2020)	√	CCT	Hubei	ST: 51 M: 28, F: 23 61.57 ± 1.84	ST: 52 M: 24, F: 28 66.35 ± 1.82	decoction+Chinese patent drug+WT1.2.6.7	WT1.2.6.7			1. CRP 2. Albumin 3. Cases number of absorption and improvement by lung CT 4. Cure rate 5. Death rate	1.P<0.01 2.P<0.05 3.P<0.05 4.P>0.05 5. Trial 21.6%; Control 30.77%	Trail: 2 cases of mild gastrointestinal reactions
	

MT, mild type; CT, common type; ST, serious type; WT, western treatment.

WT: 1. antiviral; 2. anti-infection/anti-inflammatory/antibiotics; 3. immunoregulation; 4. gastrointestinal regulation; 5. relieving cough and asthma; 6. oxygen therapy; 7. glucocorticoid; 8. nutritional support; 9. nlgesics; 10. liver protection; 11. anti-anxiety.

**Table 2:** Basic characteristics of the included studies (CS).

Included trials	Funding	Study region	Sample characteristics type; male/female; age (y)	Interventions	Duration	Fever improvement time(d)	Outcome index	Self before and after comparison	Adverse reactions
CHENG 0219 (Cheng and Li, 2020)		Hubei	CT:54 M:29.F:25 60.1 ± 16.98	Chinese patent drug+WT1.3.2.7	7 d	3.6 ± 2.14	1. Disappearance rate of fever 2. Disappearance rate of fatigue, disappearance days of fatigue 3. Disappearance rate of cough, disappearance days of cough 4. Disappearance rate of choking sensation in chest 5. Disappearance rate of anhelation 6. Disappearance rate of anorexia 7. Disappearance rate of moist rale 8. Effective rate 9. Death rate	1.80%, 2.75.7%, 4.1 ± 2.58 3.76.7%, 5.3 ± 2.63 4.84.6% 5.100% 6.40.0% 7.89.5% 8.81.6% 9. Not mentioned	No adverse response
WANG 0228 (Wang T. et al., 2020)		Jilin	MT,CT,ST:50 M:30.F:20 44.52 ± 16.12	decoction+WT1.2.6.7	7 d		1. Total effective rate 2. Disappearance rate of aversion to cold 3. Disappearance rate of thirsty 4. Disappearance rate of fever 5. Disappearance rate of sweating 6. Disappearance rate of nasal obstruction 7. Disappearance rate of headache body ache 8. Disappearance rate of short of breath 9. Disappearance rate of nausea 10. Disappearance rate of choking sensation in chest 11. Disappearance rate of diarrhea 12. Disappearance rate of anorexia 13. Disappearance rate of expectoration 14. Disappearance rate of fatigue 15. Disappearance rate of cough 16. Death rate	1.98.00% 2.100% 3.100% 4.96.96% 5.90.91% 6.73.33% 7.73.33% 8.72% 9.64.54% 10.64% 11.63.64% 12.55.56% 13.30.30% 14.25.93% 15.10.53% 16.0%	
BIN 0229 (Bin et al., 2020)	√	Hubei	MT:45 ST:10 M:31.F:24 53.9 ± 17.1	Chinese patent drug+WT1.2.6.7			1. Effective rate of mild patients 2. Effective rate of severe patients 3. Death rate	1.95.6% 2.90.0% 3.9.1%	
GONG 0309 (Gong et al., 2020)	√	Chongqing	CT:188 ST:37 M:125.F:100 0.6-82	decoction+WT1.2			1. Lymphocyte of severe patients 2. Albumin of severe patients 3. CRP of severe patients 4. CD4+,CD8+ of severe patients 5. Death rate	1.Gradually increase 2.Gradually increase 3.Drop to normal 4.Increase 5. Not mentioned	
FANG 0312 (Fang et al., 2020)	√	Hubei	MT:90 CT:98 ST:120 M:156.F:152 30-86	decoction, Chinese patent drug+WT1.2.7		5.0 ± 3.8	1. Remaining proportion of fever 2. Improvement time and remaining proportion of diarrhea 3. Improvement time and remaining proportion of choking sensation in chest 4. Improvement time and remaining proportion of fatigue 5. Improvement time and remaining proportion of cough 6. Death rate	1.0% 2.6.3 ± 3.8, 0% 3.8.5 ± 4.4,2.4% 4.7.1 ± 3.6,3.6% 5.10.4 ± 4.8,35.7% 6. Not mentioned	
ZHU 0319 (Zhu et al., 2020)		Jiangsu	CT:22 ST:1 M:10.F:13 50.0 ± 13.0	Chinese patent drug+decoction+WT1.2.6.7			1. Absolute value of LY 2. CRP 3. Improvement rate of inflammatory change absorption of lung CT 4. Time of nucleic acid test turning negative 5. Death rate	1.Obviously increase 2.Obviously decline 3.65.2% 4.11.6 ± 0.8 5. Not mentioned	
HU 0320 (Hu et al., 2020)	√	Henan	CT:19 M:8.F:11 40.55 ± 10.59	decoction+WT1.6			1. Effective rate 2. Hospitalization average time 3. Fever, cough 4. Shortness of breath, fatigue, sweating, painful abdominal mass, nausea, anorexia, diarrhea 5. Lung CT 6. Rate of turning to severe type 7. Death rate	1.100% 2.(16.36 ± 4.95)d 3.Disappear 4.Relief 5.Obvious improvement 6.0% 7. Not mentioned	
YANG 0324 (Yang Z. et al., 2020)	√	Henan	MT,CT:13 M:10,F:3 41.31 ± 13.51	decoction+WT1.2.3.4.5		3 ± 0.71	1. Improvement time of cough 2. Improvement time of fatigue 3. Improvement time of diarrhea 4. Improvement time of choking sensation in chest 5. Lung CT 6. NEUT, LY, LY/%, SCR 7. PLT, CRP, ALT, AST, TBIL, ALP, GGT, BUN, LDH 8. Death rate	1.(6 ± 2)d 2.(5 ± 1.10)d 3.(6 ± 2.12)d 4.(4 ± 1.54)d 5.Most of them still had lesions, and only 1 mild case was cured 6.P<0.05 7.P>0.05 8. Not mentioned	

MT, mild type; CT, common type; ST, serious type; WT, western treatment, WT: 1. antiviral; 2. anti-infection/anti-inflammatory/antibiotics; 3. immunoregulation; 4. gastrointestinal regulation; 5. relieving cough and asthma; 6. oxygen therapy; 7. glucocorticoid; 8. nutritional support; 9. nlgesics; 10. liver protection; 11. anti-anxiety.

### Analysis of the Law of Prescription Use in TCM

#### Frequency Analysis of Single Chinese Herbal Medicine

The statistical analysis showed that 34 traditional Chinese medicine prescriptions, involving 106 traditional Chinese medicines, were used in 20 clinical studies. The frequency of traditional Chinese medicine use was sorted. The top three drugs were *Glycyrrhizae Radix Et Rhizoma* (Gancao), *Ephedrae Herba* (Mahuang), and *Armeniacae Semen Amarum* (Kuxingren). *Ephedrae Herba* (Mahuang) aids in freeing lung, relieving cough and asthma and releasing exterior syndrome; *Armeniacae Semen Amarum* (Kuxingren) helps to depress qi and relieve cough and asthma; and *Glycyrrhizae Radix Et Rhizoma* (Gancao) facilitates in relieving cough and reducing sputum and coordinating of drugs. The three drugs are commonly used for cough and sputum and are also the basic components of Maxing Ganshi decoction in traditional Chinese medicine to treat cough and asthma. In the included prescriptions, 24 drugs were found with a frequency of ≥5 (**Table 3**). According to the traditional Chinese medicine category to sort out the 106 traditional Chinese medicines, the top 3 most frequently used are heat-clearing medicines, exterior syndrome-relieving medicines and phlegm-resolving and cough and asthma-relieving medicines, followed by damp-resolving medicines, tonify medicines, and damp-draining diuretic medicines. The details are presented in **Table 4**.

**Table 3:** Frequency of traditional Chinese herbal medicine (frequency≥5).

No.	Chinese name	Latin name	Freq.	No.	Chinese name	Latin name	Freq.
1	Gancao	*Glycyrrhizae Radix Et Rhizoma*	18	13	Renshen	*Ginseng Radix Et Rhizoma*	8
2	Mahuang	*Ephedrae Herba*	16	14	Shigao	*Gypsum Fibrosum*	8
3	Kuxingren	*Armeniacae Semen Amarum*	14	15	Taoren	*Persicae Semen*	7
4	Huangqin	*Scutellariae Radix*	12	16	Chaihu	*Bupleuri Radix*	7
5	Cangzhu	*Atractylodis Rhizoma*	12	17	Lianqiao	*Forsythiae Fructus*	7
6	Fuling	*Poria*	11	18	Huangqi	*Astragali Radix*	6
7	Banxia	*Pinelliae Rhizoma*	11	19	Yiyiren	*Coicis Semen*	6
8	Binglang	*Arecae Semen*	10	20	Dahuang	*Rhei Radix Et Rhizoma*	5
9	Chenpi	*Citri Reticulatae Pericarpium*	9	21	Baizhu	*Atractylodis Macrocephalae Rhizoma*	5
10	Houpo	*Magnoliae Officinalis Cortex*	9	22	Baishao	*Paeoniae Radix Alba*	5
11	Caoguo	*Tsaoko Fructus*	8	23	Zhimu	*Anemarrhenae Rhizoma*	5
12	Guanghuoxiang	*Pogostemonis Herba*	8	24	Chantui	*Cicadae Periostracum* *(Periostracum Cicadae Cryptotympana atrata Fabricius)*	5

**Table 4:** Frequency of types of traditional Chinese herbal medicine.

No	Types	Freq.	Types of Medicines
1	Heat-clearing medicines	65	23
2	Exterior syndrome-relieving medicines	55	16
3	Phlegm-resolving and cough and asthma-relieving medicines	48	15
4	Damp-resolving medicines	43	7
5	Tonify medicines	42	15
6	Damp-draining diuretic medicines	30	9
7	Qi-regulating medicines	23	4
8	Blood-activating and stasis-resolving medicines	11	4
9	Interior-warming medicines	8	3
10	Resolving wind-damp medicines	6	4
11	Astringent medicines	5	3
12	Purgative medicines	5	1
13	Clearing away toxin and killing parasites medicines	2	2
14	Liver-calming and wind-extinguishing medicines	1	1

The frequency of application of Glycyrrhizae Radix Et Rhizoma (gancao) has not been counted in the statistics, because of Glycyrrhizae Radix Et Rhizoma (gancao) commonly used as harmonizing herb in TCM decoctions.

#### Analysis of the Association Rules of Traditional Chinese Herbal Medicine

The association rules of traditional Chinese medicine for the included prescriptions were analyzed. The support was set to 20%. The results showed 10 associations of traditional Chinese medicine with a confidence of above 0.8. The association of traditional Chinese medicine with a confidence of 1 was *Gypsum Fibrosum* (Shigao)->*Armeniacae Semen Amarum* (Kuxingren), *Tsaoko Fructus* (Caoguo) -> *Arecae Semen* (Binglang). The association of traditional Chinese medicine with a confidence level of above 0.86 was *Gypsum Fibrosum* (Shigao) -> *Ephedrae Herba* (Mahuang), *Gypsum Fibrosum* (Shigao), *Armeniacae Semen Amarum* (Kuxingren) -> *Ephedrae Herba* (Mahuang), *Ephedrae Herba* (Mahuang), *Arecae Semen* (Binglang) -> *Atractylodis Rhizoma* (Cangzhu), *Ephedrae Herba* (Mahuang), *Arecae Semen* (Binglang) -> *Armeniacae Semen Amarum* (Kuxingren), *Atractylodis Rhizoma* (Cangzhu), *Arecae Semen* (Binglang) -> *Ephedrae Herba* (Mahuang). **Table 5** presents the analysis of specific association rules.

**Table 5:** Analysis of the association rules of traditional Chinese herbal medicine.

No.	Chinese name	Latin name	Confidence coefficient
1	Shigao -> Kuxingren	*Gypsum Fibrosum* -> *Armeniacae Semen Amarum*	1
2	Caoguo-> Binglang	*Tsaoko Fructus* -> *Arecae Semen*	1
3	Shigao -> Mahuang	*Gypsum Fibrosum* -> *Ephedrae Herba*	0.875
4	Shigao, Kuxingren -> Mahuang	*Gypsum Fibrosum*, *Armeniacae Semen Amarum* -> *Ephedrae Herba*	0.875
5	Mahuang, Binglang -> Cangzhu	*Ephedrae Herba*, *Arecae Semen* -> *Atractylodis Rhizoma*	0.875
6	Mahuang, Binglang -> Kuxingren	*Ephedrae Herba*, *Arecae Semen*-> *Armeniacae Semen Amarum*	0.875
7	Cangzhu, Binglang ->Mahuang	*Atractylodis Rhizoma*, *Arecae Semen* -> *Ephedrae Herba*	0.875
8	Kuxingren -> Mahuang	*Armeniacae Semen Amarum*-> *Ephedrae Herba*	0.857
9	Banxia -> Fuling	*Pinelliae Rhizoma* -> *Poria*	0.82
10	Fuling -> Banxia	*Poria* -> *Pinelliae Rhizoma*	0.82

#### Analysis of Chinese Herbal Medicine Combinations Network

**Figure 2:**
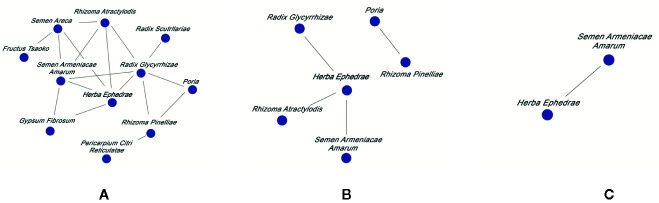
Commonly used Chinese herbal medicine combinations network diagram for NCP with different support rate. Support rate was **(A)** ≥20%, **(B)** ≥25%, and **(C)** ≥30%.

The relationship among different drug combinations was visualized using the network display function of the traditional Chinese medicine inheritance auxiliary system. The results showed that *Ephedrae Herba* (Mahuang)–*Armeniacae Semen Amarum* (Kuxingren) had the highest support, as the most common core combination, followed by *Pinelliae Rhizoma* (Banxia)–*Poria* (Fuling), *Ephedrae Herba* (Mahuang)–*Glycyrrhizae Radix Et Rhizoma* (Gancao) and *Ephedrae Herba* (Mahuang)–*Atractylodis Rhizoma* (Cangzhu). This result indicates that commonly used clinical treatments for NCP involve depressing qi, relieving cough, eliminating dampness and eliminating phlegm. The Chinese herbal medicine combinations network is presented in **Figure 2**.

### Analysis of Application of Classical Prescriptions of TCM

Studies involving the application of classical prescriptions of TCM were collected and summarized. Six studies were obtained. Among these classical prescriptions, Da Yuan decoction and Ganlu Xiaodu pill were created by doctors Wu Youke (Ming Dynasty and Ye Tianshi (Qing Dynasty) and who studied in epidemic exogenous febrile diseases, while Maxing Ganshi decoction was created by doctor Zhang Zhongjing (Han Dynasty) who researched on exogenous cold induced febrile diseases. Modern prescriptions are mostly added and subtracted by classical prescriptions. For example, the Qingfei Paidu decoction recommended by the State Administration of Traditional Chinese Medicine is based on Maxing Ganshi decoction, Shegan Mahuang decoction, Wuling powder and Xiao Chaihu decoction. The classical prescriptions with a literature frequency of ≥ 2 are presented in **Table 6**.

**Table 6:** The commonly used classical prescriptions of TCM for NCP.

No.	Classical Prescriptions of TCM	Components Latin name(Chinese name)	Source(year of completion)	Freq.	Application of cases
1	Ganlu Xiaodu Pill	*Amomi Fructus Rotundus*(Doukou), *Pogostemonis Herba*(Guanghuoxiang), *Acori Tatarinowii Rhizoma*(Shichangpu), *Menthae Haplocalycis Herba*(Bohe), *Forsythiae Fructus*(Lianqiao), *Belamcandae Rhizoma*(Shegan), *Fritillariae Cirrhosae Bulbus*(Chuanbeimu), *Scutellariae Radix* (Huangqin), *Artemisiae Scopariae Herba* (Yinchen), *Talcum*(Huashi), *Akebiae Caulis*(Mutong)	Secret of Medical Efficacy AD 1831	3	40
2	Maxing Ganshi Decoction	*Ephedrae Herba*(Mahuang), *Armeniacae Semen Amarum*(Kuxingren), *Gypsum Fibrosum*(Shigao), *Glycyrrhizae Radix Et Rhizoma* (Gancao)	Treatise on Febrile Diseases AD 200	2	80
3	Huopo Xialing Decoction	*Pogostemonis Herba* (Guanghuoxiang), *Sojae Semen Praeparatum (Dandouchi*), *Amomi Fructus Rotundus* (Doukou), *Magnoliae Officinalis Cortex*(Houpo), *Pinelliae Rhizoma* (Banxia), *Armeniacae Semen Amarum*(Kuxingren), *Poria* (Fuling), *Polyporus*(Zhuling), A*lismatis Rhizoma*(Zexie), *Coicis Semen* (Yiyiren)	Original Medical Theory AD 1861	2	45
4	Da Yuan Decoction	*Arecae Semen* (Binglang), *Magnoliae Officinalis Cortex* (Houpo), *Tsaoko Fructus* (Caoguo), *Anemarrhenae Rhizoma*(Zhimu), *Paeoniae Radix Alba*(Baishao), *Scutellariae Radix* (Huangqin), *Glycyrrhizae Radix Et Rhizoma*(Gancao)	Treatise on Acute Epidemic Febrile Diseases AD 1642	2	42
5	Haoqin Qingdan Decoction	*Artemisiae Annuae Herba*(Qinghao), *Bambusae Caulis In Taenias*(Zhuru), *Pinelliae Rhizoma*(Banxia), *Poria* (Fuling), *Scutellariae Radix* (Huangqin), *Aurantii Fructus*(Zhiqiao), *Citri Reticulatae Pericarpium* (Chenpi), *Talcum*(Huashi), Indigo Naturalis(Qingdai), *Glycyrrhizae Radix Et Rhizoma*(Gancao)	Revisiting of Treatise on Acute Epidemic Febrile Diseases AD 1956	2	25
6	Xuanbai Chengqi Decoction	*Gypsum Fibrosum* (Shigao), *Rhei Radix Et Rhizoma* (Dahuang), *Armeniacae Semen Amarum*(Kuxingren), *Trichosanthis Fructus* (Gualou)	Item Differentiation of Warm Febrile Diseases AD 1798	2	18
7	Tingli Dazao Xiefei Decoction	*Descurainiae Semen Lepidii Semen* (Tinglizi), *Jujubae Fructus*(Dazao)	Synopsis of Golden Chamber AD 200	2	18

### Analysis of Application of Chinese Patent Medicine

Given its convenient application, Chinese patent medicine has gained increasing research attention. An analysis of the use of Chinese patent medicine in 20 clinical studies showed that Lianhua Qingwen capsules/granules are the most widely used. These capsules have been widely studied to verify their clinical efficacy. Lianhua Qingwen can act on coronavirus through multiple components, targets and pathways *via* their broad-spectrum antiviral, antibacterial and antipyretic; cough relief; sputum reduction and immune regulation effects (Ling et al., 2020). In the treatment of NCP, Xuebijing and other traditional Chinese medicine injections have been used several times. Xuebijing can antagonize endotoxins (Zhang, 2018; Wang, 2019) and inhibit the excessive release of inflammatory mediators, such as interferon and interleukin (Tian et al., 2019), thereby inhibiting inflammation and enhancing immunity (Diao et al., 2015). The academician Zhang Boli emphasized that the early application of traditional Chinese medicine injection can play a vital role in treating critical patients (Tencent news, 2020b). **Table 7** presents The commonly used Chinese patent medicine for NCP.

**Table 7:** The commonly used Chinese patent medicine for NCP.

No.	Chinese patent medicine	Components Latin name(Chinese name)	Freq.	Prop.
1	Lianhua Qingwen capsule/granule	*Forsythiae Fructus*(Lianqiao), *Lonicerae Japonicae Flos* (Jinyinhua), *Ephedrae Herba*(Mahuang), *Armeniacae Semen Amarum*(Kuxingren), *Gypsum Fibrosum* (Shigao), *Isatidis Radix*(Banlangen), *Dryopteridis Crassirhizomatis Rhizoma*(Mianma Guanzhong), *Houttuyniae Herba*(Yuxingcao), *Pogostemonis Herba* (Guanghuoxiang), *Rhei Radix Et Rhizoma*(Dahuang), *Rhodiolae Crenulatae Radix Et Rhizoma*(Hongjingtian)	7	35.00%
2	Xue Bi Jing Injection	*Carthami Flos*(Honghua), *Paeoniae Radix Rubra* (Chishao), *Chuanxiong* *Rhizoma* (Chuanxiong), *Salviae Miltiorrhizae Radix Et Rhizoma* (Danshen), *Angelicae Sinensis Radix*(Danggui)	3	15.00%
3	Shufeng Jiedu Capsule	*Polygoni Cuspidati Rhizoma Et Radix* (Huzhang), *Forsythiae Fructus* (Lianqiao), *Isatidis Radix* (Banlangen), *Bupleuri Radix* (Chaihu), *Herba Patriniae*(Baijiangcao), *Verbenae Herba* (Mabiancao), *Phragmitis Rhizoma* (Lugen), *Glycyrrhizae Radix Et Rhizoma* (Gancao)	3	15.00%

### Investigation of the Observation Indicators

In 20 studies on the treatment of NCP, the most commonly used clinical observation and evaluation indices was fever improvement time, followed by cough improvement time, shortness of breath improvement time, chest CT, and TCM syndrome scale score. Some articles also used the disappearance rate of other accompanying symptoms and CRP examination as observation indices. From **Table 1**, we can see the fever improvement time in the trial group was significantly shorter than that in the control group. In **Table 8**, we listed the Chinese name, Latin name in Chinese pharmacopeia, and Name in Medicinal Plant Names Services.

**Table 8:** Drug name comparison table.

No.	Chinese name	Latin name in Chinese pharmacopeia	Name in Medicinal Plant Names Services (MPNS)
1	Baijiangcao	*Herba Patriniae*	Patrinia scabiosifolia Link
2	Baishao	*Paeoniae Radix Alba*	Paeonia lactiflora Pall.
3	Baizhi	*Angelicae Dahuricae Radix*	Angelica dahurica (Hoffm.) Benth. & Hook.f. ex Franch. & Sav.
4	Baizhu	*Atractylodis Macrocephalae Rhizoma*	Atractylodes macrocephala Koidz.
5	Banlangen	*Isatidis Radix*	Isatis tinctoria L.
6	Banxia	*Pinelliae Rhizoma*	Pinellia ternata (Thunb.) Makino
7	Binglang	*Arecae Semen*	Areca catechu L.
8	Bohe	*Menthae Haplocalycis Herba*	Mentha canadensis L.
9	Cangzhu	*Atractylodis Rhizoma*	Atractylodes lancea (Thunb.) DC.
10	Caoguo	*Tsaoko Fructus*	Lanxangia tsao-ko (Crevost & Lemarié) M.F.Newman & Skornick.
11	Chaihu	*Bupleuri Radix*	Bupleurum chinense DC.
12	Chantui	*Cicadae Periostracum (Periostracum Cicadae Cryptotympana atrata Fab- ricius)*	——
13	Chenpi	*Citri Reticulatae Pericarpium*	Citrus × aurantium L.
14	Chishao	*Paeoniae Radix Rubra*	Paeonia anomala subsp. veitchii (Lynch) D.Y.Hong & K.Y.Pan
15	Chuanbeimu	*Fritillariae Cirrhosae Bulbus*	Fritillaria cirrhosa D.Don
16	Chuanxiong	*Chuanxiong Rhizoma*	Conioselinum anthriscoides 'Chuanxiong'
17	Dahuang	*Rhei Radix Et Rhizoma*	Rheum palmatum L.
18	Dandouchi	*Sojae Semen Praeparatum*	Glycine max (L.) Merr.
19	Danggui	*Angelicae Sinensis Radix*	Angelica sinensis (Oliv.) Diels
20	Danshen	*Salviae Miltiorrhizae Radix Et Rhizoma*	Salvia miltiorrhiza Bunge
21	Daqingye	*Isatidis Folium*	Isatis tinctoria L.(Folium Isatidis)
22	Dazao	*Jujubae Fructus*	Ziziphus jujuba Mill.
23	Dihuang	*Rehmanniae Radix*	Rehmannia glutinosa (Gaertn.) DC.
24	Doukou	*Amomi Fructus Rotundus*	Alpinia hainanensis K.Schum.
25	Fangfeng	*Saposhnikoviae Radix*	Saposhnikovia divaricata (Turcz. ex Ledeb.) Schischk.
26	Fengfang	*Vespae Nidus*	——
27	Fuling	*Poria*	Smilax glabra Roxb. ( Poria cocos (Schw. ) Wolf.)
28	Fuzi	*Aconiti Lateralis Radix Praeparata*	Aconitum carmichaeli Debeaux (Radix Aconiti Lateralis Preparata)
29	Gancao	*Glycyrrhizae Radix Et Rhizoma*	Glycyrrhiza uralensis Fisch. ex DC.
30	Ganjiang	*Zingibneris Rhizoma*	Zingiber officinale Roscoe (Rhizoma Zingiberis)
31	Gegen	*Puerariae Lobatae Radix*	Pueraria montana var. lobata (Willd.) Maesen & S.M.Almeida ex Sanjappa & Predeep
32	Gualou	*Trichosanthis Fructus*	Trichosanthes kirilowii Maxim.
33	Guanghuoxiang	*Pogostemonis Herba*	Pogostemon cablin (Blanco) Benth.
34	Guizhi	*Cinnamomi Ramulus*	Cinnamomum cassia (L.) J.Presl
35	Honghua	*Carthami Flos*	Carthamus tinctorius L.
36	Hongjingtian	*Rhodiolae Crenulatae Radix Et Rhizoma*	Rhodiola crenulata (Hook.f. & Thomson) H.Ohba
37	Hongshen	*Ginseng Radix Et Rhizoma Rubra*	Panax ginseng C.A.Mey.
38	Houpo	*Magnoliae Officinalis Cortex*	Magnolia officinalis Rehder & E.H.Wilson
39	Huanglian	*Coptidis Rhizoma*	Coptis chinensis Franch.
40	Huangqi	*Astragali Radix*	Astragalus mongholicus Bunge
41	Huangqin	*Scutellariae Radix*	Scutellaria baicalensis Georgi
42	Huashi	*Talcum*	——
43	Huzhang	*Polygoni Cuspidati Rhizoma Et Radix*	Reynoutria japonica Houtt.
44	Jiangcan	*Bombyx Batryticatus*	——
45	Jianghuang	*Curcumae Longae Rhizoma*	Curcuma longa L.
46	Jinyinhua	*Lonicerae Japonicae Flos*	Lonicera japonica Thunb.
47	Kuxingren	*Armeniacae Semen Amarum*	Prunus armeniaca L.
48	Lianqiao	*Forsythiae Fructus*	Forsythia suspensa (Thunb.) Vahl
49	Lugen	*Phragmitis Rhizoma*	Phragmites australis subsp. australis
50	Mabiancao	*Verbenae Herba*	Verbena officinalis L.
51	Mahuang	*Ephedrae Herba*	Ephedra sinica Stapf
52	Maidong	*Ophiopogonis Radix*	Ophiopogon japonicus (Thunb.) Ker Gawl.
53	Mianma Guanzhong	*Dryopteridis Crassirhizomatis Rhizoma*	Dryopteris crassirhizoma Nakai
54	Moyao	*Myrrha*	Commiphora myrrha (T.Nees) Engl.
55	Mudanpi	*Moutan Cortex*	Paeonia × suffruticosa Andrews
56	Mutong	*Akebiae Caulis*	Akebia quinata (Thunb. ex Houtt.) Decne.
57	Niubangzi	*Arctii Fructus*	Arctium lappa L.
58	Pugongying	*Taraxaci Herba*	Taraxacum mongolicum Hand.-Mazz.
59	Qianhu	*Peucedani Radix*	Kitagawia praeruptora (Dunn) Pimenov
60	Qingdai	*Indigo Naturalis*	Persicaria tinctoria (Aiton) Spach
61	Qinghao	*Artemisiae Annuae Herba*	Artemisia annua L.
62	Renshen	*Ginseng Radix Et Rhizoma*	Panax ginseng C.A.Mey.
63	Sangbaipi	*Mori Cortex*	Morus alba L.
64	Shancigu	*Cremastrae Pseudobulbus Pleiones Pseudobulbus*	Pleione yunnanensis (Rolfe) Rolfe
65	Shegan	*Belamcandae Rhizoma*	Iris domestica (L.) Goldblatt & Mabb.
66	Shengjiang	*Zingiberis Rhizoma Recens*	Zingiber officinale Roscoe
67	Shengma	*Cimicifugae Rhizoma*	Actaea cimicifuga L.
68	Shichangpu	*Acori Tatarinowii Rhizoma*	Acorus calamus var. angustatus Besser
69	Shigao	*Gypsum Fibrosum*	——
70	Taizishen	*Pseudostellariae Radix*	Pseudostellaria heterophylla (Miq.) Pax
71	Taoren	*Persicae Semen*	Prunus persica (L.) Batsch
72	Tinglizi	*Descurainiae Semen Lepidii Semen*	Descurainia sophia (L.) Webb ex Prantl
73	Weilingxian	*Clematidis Radix Et Rhizoma*	Clematis chinensis Osbeck
74	Wumei	*Mume Fructus*	Prunus mume (Siebold) Siebold & Zucc.
75	Wuweizi	*Schisandrae Chinensis Fructus*	Schisandra chinensis (Turcz.) Baill.
76	Xinyi	*Magnoliae Flos*	Magnolia biondii Pamp.
77	Xixiancao	*Siegesbeckiae Herba*	Sigesbeckia orientalis L.
78	Xixin	*Asari Radix Et Rhizoma*	Asarum sieboldii Miq.
79	Xuanshen	*Scrophulariae Radix*	Scrophularia ningpoensis Hemsl.
80	Yinchen	*Artemisiae Scopariae Herba*	Artemisia capillaris Thunb.
81	Yiyiren	*Coicis Semen*	Coix lacryma-jobi var. ma-yuen (Rom.Caill.) Stapf
82	Yuxingcao	*Houttuyniae Herba*	Houttuynia cordata Thunb.
83	Zexie	*Alismatis Rhizoma*	Alisma plantago-aquatica subsp. orientale (Sam.) Sam.
84	Zhebeimu	*Fritiliariae Thunbergil Bulbus*	Fritillaria thunbergii Miq.
85	Zhimu	*Anemarrhenae Rhizoma*	Anemarrhena asphodeloides Bunge
86	Zhiqiao	*Aurantii Fructus*	Citrus trifoliata L.
87	Zhuling	*Polyporus (Polyporus umbellatus(Pers.) Fr.)*	——
88	Zhuru	*Bambusae Caulis In Taenias*	Bambusa beecheyana Munro
89	Ziwan	*Asteris Radix Et Rhizoma*	Aster tataricus L.f.

The drugs were listed in the order of their Chinese name.

The top frequency that search term appeared in medicinal plant literature was chosen.

—— MPNS could not match the search term.

## Discussion

On the discussion of epidemic, the ancient Chinese doctor Wu Youke from the Ming Dynasty pointed out it was caused by epidemic pathogenic evils. Given its strong infectivity, disease location and clinical characteristics, NCP can be named “pulmonary epidemic disease” (Guo and Wan, 2020). The main consensus regarding its pathogenesis is that the virus invades the lungs and causes vital qi deficiency. The pathological nature is dampness, heat, toxin, deficiency and stasis.

This study mainly uses bibliometrics and data mining methods to obtain a systematic summary of clinical studies published at this stage and systematically analyses the published information, research design, intervention measures and observation indicators. A summary of the research methods indicates that only 2 RCTs were conducted. Most of the studies were CCTs and CSs. Considering the large number of patients and the rapid spread of the epidemic, the shortage of medical resources has led to the unconditional implementation of RCT research. The treatment of patients is the first priority at this time.

Regarding the time distribution of publications, the time that research on traditional Chinese medicine treatment of NCP was conducted synchronized with the epidemic. Furthermore, the symptom improvement rate and symptom scores in the observation and evaluation indicators fully reflect the characteristics of the judgment standard of clinical efficacy of traditional Chinese medicine. The total number of observation cases also reflects the high participation of traditional Chinese medicine in this anti-epidemic treatment. A clear understanding of Chinese herbal medicines use has been achieved through the data mining and analysis of prescriptions for treatment of NCP. In addition to *Glycyrrhizae Radix Et Rhizoma* (Gancao), *Ephedrae Herba* (Mahuang), *Armeniacae Semen Amarum* (Kuxingren) *Atractylodis Rhizoma* (Cangzhu) and *Scutellariae Radix* (Huangqin) are frequently used. An analysis of drug categories showed that heat-clearing medicine, exterior syndrome-relieving medicines, phlegm-resolving and cough and asthma-relieving medicines, and humidifying drugs are frequently used. This finding suggests that dampness and toxin accumulating in the lung are the main pathogenesis of NCP. *Ephedrae Herba* (Mahuang)-*Armeniacae Semen Amarum* (Kuxingren) had the highest support and high confidence in the association rules, which reflects the classic compatibility of Maxing Shigan decoction. About the high frequency Chinese herbal medicines, most of it enters the lung meridian or spleen meridian. Chinese medicine recognizes that NCP mainly involves the lung. The spleen is the source of phlegm, and the lung is the sputum storage position, phlegm and dampness caused by lung and spleen disease. The results of clinical application analysis of Chinese patent medicines reflect the participation in clinical treatment. Given their wide range of applications and convenient application, Chinese patent medicines play an important role in clinical treatment of the epidemic in China. Traditional Chinese medicine for treatment of NCP is worthy of global attention.

Our study has several limitations. Randomized controlled trials are the most commonly used to judge the effectiveness of interventions. This review only included two RCTs. And they did not mention blinding method. In addition, the interventions, treatment courses, and observation indicators of each study were quite different, so meta-analysis cannot be done. High-quality RCTs on the effectiveness and safety of traditional Chinese medicine in the treatment of new coronary pneumonia need further study.

## Author Contributions

ZZ conceived and wrote the manuscript draft. SF designed the study and revised the manuscript. NG drafted the manuscript. YW was responsible for data collection. PC helped data management. YT was in charge of statistical analysis of data. All authors contributed to the article and approved the submitted version.

## Funding

We are very grateful for the financial support from the Special Research Project of Traditional Chinese Medicine Industry (201107006) and the School-level scientific research project of Tianjin University of Traditional Chinese Medicine (XJ201801).

## Conflict of Interest

The authors declare that the research was conducted in the absence of any commercial or financial relationships that could be construed as a potential conflict of interest.

## Abbreviations

NCP, new coronary pneumonia; CCT, clinical control trials; RCT, randomized controlled trials; CS, case series studies; TCM, Traditional Chinese medicine.
