# Supplementary material for: Clinical Studies on the Treatment of Novel Coronavirus Pneumonia With Traditional Chinese Medicine—A Literature Analysis
Source: Front Pharmacol. 2020 Sep 10;11:560448. doi: 10.3389/fphar.2020.560448 (PMC7511712; doi:10.3389/fphar.2020.560448)
Supplement: Supplementary file 1 [file DataSheet_1.doc]

The commonly used prescriptions of TCM for NCP

| No. | Classical Prescriptions of TCM | Components Latin name(Chinese name) dosage | Preparation, usage |
| --- | --- | --- | --- |
| 1 | Shidu Yunfei Prescription (Xia et al., 2020) | *Armeniacae Semen Amarum*(Kuxingren) 15 g  *Talcum*(Huashi) 30 g  *Atractylodis Rhizoma*(Cangzhu) 30 g  *Angelicae Dahuricae Radix*(Baizhi) 10 g  *Pinelliae Rhizoma* (Banxia) 15 g  *Pogostemonis Herba*(Guanghuoxiang) 15 g  *Poria* (Fuling) 30 g  *Ephedrae Herba*(Mahuang) 9 g  *Rhei Radix Et Rhizoma*(Dahuang) 10 g  *Cicadae Perioetracum*(Chantui) 10 g  *Arctii Fructus*(Niubangzi) 15 g  *Glycyrrhizae Radix Et Rhizoma* (Gancao) 10 g | Decoction  Half dose bid po |
| 2 | Yidu Bifei Prescription (Xia et al., 2020) | *Armeniacae Semen Amarum*(Kuxingren) 10 g  *Gypsum Fibrosum* (Shigao) 30 g  *Trichosanthis Fructus*(Gualou) 30 g  *Rhei Radix Et Rhizoma(Dahuang)6 g*  *Ephedrae Herba*(Mahuang) 12 g  *Descurainiae Semen Lepidii Semen*(Tinglizi)10g  *Persicae Semen*(Taoren) 10 g  *Tsaoko Fructus*(Caoguo) 6 g  *Arecae Semen*(Binglang) 10 g  *Atractylodis Rhizoma*(Cangzhu) 10 g | Decoction  Half dose bid po |
| 3 | Qingfei Touxie Fuzheng Prescription (Ding et al., 2020) | *Ephedrae Herba*(Mahuang) 6 g  *Gypsum Fibrosum* (Shigao) 20 g  *Armeniacae Semen Amarum*(Kuxingren) 10 g  *Lonicerae Japonicae Flos* (Jinyinhua) 30 g  *Forsythiae Fructus*(Lianqiao) 15 g  *Phragmitis Rhizoma* (Lugen) 30 g  *Coicis Semen* (Yiyiren) 30 g  *Bombyx Batryticatus*(Jiangcan) 10 g  *Cicadae Perioetracum*(Chantui) 10 g  *Polygoni Cuspidati Rhizoma Et Radix* (Huzhang) 15 g  *Curcumae Longae Rhizoma*(Jianghuang) 10 g  *Paeoniae Radix Alba*(Baishao) 10 g  *Pseudostellariae Radix*(Taizishen) 20 g  *Glycyrrhizae Radix Et Rhizoma* (Gancao) 15 g | Decoction  Half dose bid po |
| 4 | Toujie Quwen Granule Prescription (Fu et al., 2020[2020-04-22) | *Forsythiae Fructus*(Lianqiao)30 g  *Cremastrae Pseudobulbus Pleiones Pseudobulbus*(Shancigu) 20 g  *Lonicerae Japonicae Flos* (Jinyinhua) 15 g  *Scutellariae Radix* (Huangqin) 10 g  *Isatidis Folium*(Daqingye) 10 g  *Bupleuri Radix* (Chaihu) 5 g  *Artemisiae Annuae Herba*(Qinghao) 10 g  *Cicadae Perioetracum*(Chantui) 10 g  *Peucedani Radix*(Qianhu) 5 g  *Fritillariae Cirrhosae Bulbus*(Chuanbeimu) 10 g  *Fritiliariae Thunbergil Bulbu*s(Zhebeimu) 10 g  *Mume Fructus*(Wumei) 30 g  *Scrophulariae Radix*(Xuanshen) 10 g  *Astragali Radix*(Huangqi) 45 g  *Poria* (Fuling) 30 g  *Pseudostellariae Radix*(Taizishen) 15 g | Granule, [infused with boiled water](http://dict.cnki.net/dict_result.aspx?scw=冲服&tjType=sentence&style=&t=infused+with+water)  Half dose bid po |
| 5 | Buzhong Yiqi Prescription (Wang et al., 2020) | *Astragali Radix*(Huangqi) 30g  *Ginseng Radix Et Rhizoma*(Renshen)15g  *Glycyrrhizae Radix Et Rhizoma* (Gancao) 15g  *Atractylodis Macaocephalae Rhizoma*(Baizhu) 10g  *Citri Reticulatae Pericarpium* (Chenpi)6g  *Angelicae Sinensis Radix*(Danggui) 10g  *Jujubae Fructus*(Dazao) 6g  *Zingiberis Rhizoma Recens*(Shengjiang) 9pieces  *Bupleuri Radix* (Chaihu) 12g  *Cimicifugae Rhizoma*(Shengma) 6g | Decoction  Half dose bid po |
| 6 | Huhuang Paidu Cream (Wang et al., 2020) | *Coptidis Rhizama*(Huanglian) 20g  *Rhei Radix Et Rhizoma*(Dahuang) 10g  *Scutellariae Radix* (Huangqin) 10g  *Atractylodis Rhizoma*(Cangzhu) 10g  *Asteris Radix Et Rhizoma*(Ziwan) 10g  *Houttuyniae Herba*(Yuxingcao) 10g  *Taraxaci Herba*(Pugongying) 10g  *Polygoni Cuspidati Rhizoma Et Radix* (Huzhang) 10g  *Astragali Radix*(Huangqi)20g | Decoction  Half dose bid po |
| 7 | Baimu Qingre Jiedu Cream (Wang et al., 2020) | *Puerariae Lobatae Radix*(Gegen) 15g  *Angelicae Dahuricae Radix*(Baizhi)12g  *Magnoliae Flos*(Xinyi) 9g  *Isatidis Radix*(Banlangen) 30g  *Forsythiae Fructus*(Lianqiao) 15g  *Fritiliariae Thunbergil Bulbus*(Zhebeimu)12g | Decoction  Half dose bid po |
| 8 | Yidu Hanshi Bifei Prescription (Wang et al.,2020) | *Ephedrae Herba*(Mahuang) 12 g  *Paeoniae Radix Alba*(Baishao) 15 g  *Cinnamomi Ramulus*(Guizhi) 15 g  *Asari Radix Et Rhizoma*(Xixin) 3 g  *Pinelliae Rhizoma* (Banxia) 7 g  *Glycyrrhizae Radix Et Rhizoma* (Gancao) 10 g  *Schisandrae Chinensis Fructus*(Wuweizi) 10 g  *Zingibneris Rhizoma*(Ganjiang) 10 g  *Persicae Semen*(Taoren) 10 g  *Myrrha*(Moyao) 10 g  *Siegesbeckiae Herba*(Xixiancao) 30 g  *Clematidis Radix Et Rhizoma*(Weilingxian) 15 g  *Vespae Nidus*(Fengfang) 5 g  *Atractylodis Rhizoma*(Cangzhu) 30 g  *Magnoliae Officinalis Cortex*(Houpo) 15 g | Decoction  Half dose bid po |
| 9 | Severe Pneumonia Prescription (Wang et al.,2020) | *Ginseng Radix Et Rhizoma*(Renshen) 10 g  *Aconiti Lateralis Radix Praeparata*(Fuzi) 7 g  *Zingibneris Rhizoma*(Ganjiang) 20 g  *Atractylodis Macaocephalae Rhizoma*(Baizhu) 30 g  *Glycyrrhizae Radix Et Rhizoma* (Gancao) 10 g  *Coicis Semen* (Yiyiren) 50 g  *Herba Patrinia*e(Baijiangcao) 30 g  *Phragmitis Rhizoma* (Lugen) 50 g  *Persicae Semen*(Taoren) 10 g  *Paeoniae Radix Rubra* (Chishao) 20 g | Decoction  Half dose bid po |
| 10 | Maxing Ganshi Decoction (Gong et al., 2020) | *Ephedrae Herba*(Mahuang)9g  *Armeniacae Semen Amarum*(Kuxingren)9g  *Gypsum Fibrosum*(Shigao)19g  *Glycyrrhizae Radix Et Rhizoma* (Gancao)6g | Decoction  Half dose bid po |
| 11 | Huopo Xialing Decoction (Gong et al., 2020) | *Pogostemonis Herba* (Guanghuoxiang) 6g  *Sojae Semen Praeparatum* (Dandouchi) 9g  *Amomi Fructus Rotundus* (Doukou) 3g  *Magnoliae Officinalis Cortex*(Houpo) 3g  *Pinelliae Rhizoma* (Banxia) 4.5g  *Armeniacae Semen Amarum*(Kuxingren) 9g  *Poria* (Fuling) 9g  *Polyporus*(Zhuling) 9g  *Alismatis Rhizoma*(Zexie) 4.5g  *Coicis Semen* (Yiyiren) 12g | Decoction  Half dose bid po |
| 12 | Haoqin Qingdan Decoction (Gong et al., 2020) | *Artemisiae Annuae Herba*(Qinghao) 6g  *Bambusae Caulis In Taenias* (Zhuru) 9g  *Pinelliae Rhizoma*(Banxia) *4.5g*  *Poria* (Fuling) 9g  *Scutellariae Radix* (Huangqin) 9g  *Aurantii Fructus*(Zhiqiao) 4.5g  *Citri Reticulatae Pericarpium* (Chenpi) 4.5g  *Talcum*(Huashi) *3g*  *Indigo Naturalis*(Qingdai) 3g  *Glycyrrhizae Radix Et Rhizoma*(Gancao) 3g | Decoction  Half dose bid po |
| 13 | Maxingyigan Decoction, and  Huopoxialing Prescription (Hu et al., 2020) | *Ephedrae Herba*(Mahuang)6g  *Coicis Semen* (Yiyiren)30g  *Armeniacae Semen Amarum*(Kuxingren)12g  *Pogostemonis Herba*(Guanghuoxiang)12g  *Atractylodis Rhizoma*(Cangzhu)15g  *Poria* (Fuling)20g  *Magnoliae Officinalis Corte*x(Houpo)15g  *Arecae Semen*(Binglang)10g  *Tsaoko Fructus*(Caoguo)10g  *Pinelliae Rhizoma* (Banxia)15g  *Zingiberis Rhizoma Recens*(Shengjiang)10g | Decoction  One third dose tid po |
| 14 | Maxingganshi Decoction, Dayuan Decoction, and Xiaochaihu Prescription (Hu et al., 2020) | *Ephedrae Herba*(Mahuang)9g  *Armeniacae Semen Amarum*(Kuxingren) 12g  *Gypsum Fibrosum* (Shigao) 30g  *Glycyrrhizae Radix Et Rhizoma* (Gancao) 6g  *Arecae Semen*（Binglang）10g  *Tsaoko Fructus*（Caoguo）10g  *Magnoliae Officinalis Cortex*(Houpo) 10g  *Anemarrhenae Rhizoma*(Zhimu) 10g  *Scutellariae Radix* (Huangqin) 10g  *Bupleuri Radix* (Chaihu) 18g  *Paeoniae Radix Alba*(Baishao) 15g  *Forsythiae Fructus(*Lianqiao) 15g  *Atractylodis Rhizoma*(Cangzhu) 10g | Decoction  One third dose tid po |
| 15 | Xuanbaichengqi Decoction, Tinglidazaoxiefei Decoction, and Jieduhuoxue Prescription (Hu et al., 2020) | *Ephedrae Herba*(Mahuang)9g  *Armeniacae Semen Amarum*(Kuxingren)12g  *Gypsum Fibrosum* (Shigao) 30g  *Rhei Radix Et Rhizoma*(Dahuang) 10g  *Trichosanthis Fructus*(Gualou) 30g  *Persicae Semen*(Taoren) 20g  *Paeoniae Radix Rubra* (Chishao) 15g  *Descurainiae Semen Lepidii Semen*(Tinglizi) 15g  *Tsaoko Fructus*(Caoguo) 10g  *Arecae Semen*(Binglang) 10g  *Scutellariae Radix* (Huangqin) 15g  *Mori Cortex*(Sangbaipi) 15g  *Moutan Cortex*(Mudanpi) 12g  *Rehmanniae Radix*(Dihuang) 20g | Decoction  One third dose tid po |
| 16 | Ganlu Xiaodu Pill (Xia et al., 2020) | *Amomi Fructus Rotundus(Doukou) 6g*  *Pogostemonis Herba(Guanghuoxiang) 6g*  *Acori Tatarinowii Rhizoma(Shichangpu) 6g*  *Menthae Haplocalycis Herba(Bohe) 6g*  *Forsythiae Fructus(Lianqiao) 10g*  *Belamcandae Rhizoma(Shegan) 10g*  *Fritillariae Cirrhosae Bulbus (Chuanbeimu) 10g*  *Scutellariae Radix (Huangqin) 10g*  *Artemisiae Scopariae Herba (Yinchen) 10g*  *Talcum(Huashi) 20g*  *Akebiae Caulis(Mutong) 6g* | Decoction  Half dose bid po |

The commonly used Chinese patent medicine for NCP

| No. | Chinese patent medicine | Components Latin name(Chinese name) dosage/dosage ratio | Preparation, dosage |
| --- | --- | --- | --- |
| 1 | Lianhua Qingwen Granule (Yao et al., 2020; Lv  et al., 2020; Shi et al., 2020; Cheng et al., 2020; Cheng and Li, 2020) | *Forsythiae Fructus*(Lianqiao)170g  *Lonicerae Japonicae Flos* (Jinyinhua)170g  *Ephedrae Herba*(Mahuang)57g  *Armeniacae Semen Amarum*(Kuxingren)57g  *Gypsum Fibrosum* (Shigao)170g  *Isatidis Radix*(Banlangen)170g  *Dryopteridis Crassirhizomatis Rhizoma*(Mianma Guanzhong)170g  *Houttuyniae Herba*(Yuxingcao)170g  *Pogostemonis Herba* (Guanghuoxiang)57g  *Rhei Radix Et Rhizoma*(Dahuang)34g  *Rhodiolae Crenulatae Radix Et Rhizoma*(Hongjingtian)57g | Granule  Made into 1000g  6g tid po |
| 2 | Lianhua Qingwen Capsule (Bin et al., 2020; Fang et al., 2020) | *Forsythiae Fructus*(Lianqiao)255g  *Lonicerae Japonicae Flos* (Jinyinhua)255g  *Ephedrae Herba*(Mahuang)85g  *Armeniacae Semen Amarum*(Kuxingren)85g  *Gypsum Fibrosum* (Shigao)255g  *Isatidis Radix*(Banlangen)255g  *Dryopteridis Crassirhizomatis Rhizoma*(Mianma Guanzhong)255g *Houttuyniae Herba*(Yuxingcao)255g  *Pogostemonis Herba*(Guanghuoxiang)85g  *Rhei Radix Et Rhizoma*(Dahuang)51g  *Rhodiolae Crenulatae Radix Et Rhizoma*(Hongjingtian)85g | Capsule  Made into 1000 capsules  4 capsules tid po |
| 3 | Xue Bi Jing Injection (Yang et al., 2020; Fang  et al., 2020; Zhu et al., 2020) | *Carthami Flos*(Honghua) 20%  *Paeoniae Radix Rubra* (Chishao) 20%  *Chuanxiong Rhizoma* (Chuanxiong) 20%  *Salviae Miltiorrhizae Radix Et Rhizoma* (Danshen) 20%  *Angelicae Sinensis Radix*(Danggui) 20% | [I](../../../../C:/Users/lenovo/AppData/Local/youdao/dict/Application/8.9.4.0/resultui/html/index.html#/javascript:;)ntravenous [injection](../../../../C:/Users/lenovo/AppData/Local/youdao/dict/Application/8.9.4.0/resultui/html/index.html#/javascript:;)  50ml bid  iv drip |
| 4 | Shufeng Jiedu Capsule (Qv et al., 2020; Shi et al., 2020; Xiao et al., 2020) | *Polygoni Cuspidati Rhizoma Et Radix* (Huzhang)19.2%  *Forsythiae Fructus (Lianqiao), Isatidis Radix* (Banlangen*)*15.4%  *Bupleuri Radix* (Chaihu)15.4%  *Herba Patriniae*(Baijiangcao)15.4%  *Verbenae Herba* (Mabiancao)15.4%  *Phragmitis Rhizoma* (Lugen)11.5%  *Glycyrrhizae Radix Et Rhizoma* (Gancao)7.7% | Capsule  4 capsules tid po |
| 5 | Shuang Huang Lian Oral Liquid (Shi et al.,2020) | *Lonicerae Japonicae Flos* (Jinyinhua)375g  *Scutellariae Radix* (Huangqin)375g  *Forsythiae Fructus*(Lianqiao)750g | Oral liquid  Made into 500ml  10ml tid po |
| 6 | Yu Pingfeng Granule (Shi et al., 2020) | *Astragali Radix*(Huangqi)600g  *Saposhnikoviae Radix*(Fangfeng)200g  *Atractylodis Macaocephalae Rhizoma*(Baizhu)200g | Granule,  Made into 500g  5g tid po |
| 7 | Jinhua Qinggan Granule (Duan et al., 2020) | *Lonicerae Japonicae Flos* (Jinyinhua)33.3%  *Gypsum Fibrosum* (Shigao)66.7%  *Ephedrae Herba*(Mahuang)13.3%  *Armeniacae Semen Amarum*(Kuxingren)20%  *Forsythiae Fructus*(Lianqiao)33.3%  *Fritiliariae Thunbergil Bulbus*(Zhebeimu)22.2%  *Scutellariae Radix* (Huangqin)33.3%  *Anemarrhenae Rhizoma*(Zhimu)22.2%  *Menthae Haplocalycis Herba(*Bohe)13.3%  *Glycyrrhizae Radix Et Rhizoma* (Gancao)22.2%  *Arctii Fructus*(Niubangzi)33.3%  *Artemisiae Annuae Herba*(Qinghao)33.3% | Granule  6g bid po |
| 8 | Sheng Mai Injection (Yang et al., 2020) | *Ginseng Radix Et Rhizoma Rubra*(Hongshen) 100g  *Ophiopogonis Radix*(Maidong) 312g  *Schisandrae Chinensis Fructus*(Wuweizi) 156g | Injection  Made into 1000ml  20-60ml qd iv drip |

Note: bid, bis in die; po, peros; tid, ter in die; iv drip, intravenous drip; qd, quaque die.
